# Review of Evidence Supporting 2022 US Food and Drug Administration Drug Approvals

**DOI:** 10.1001/jamanetworkopen.2023.27650

**Published:** 2023-08-08

**Authors:** Robert M. Kaplan, Amanda J. Koong, Veronica Irvin

**Affiliations:** 1 Clinical Excellence Research Center, Stanford University, Stanford, California; 2 Medical student, McGovern School of Medicine, Houston, Texas; 3 College of Public Health and Human Services, Oregon State University, Corvallis

## Abstract

This cross-sectional study examines the design and funding of studies, nd evidence available on ClinicalTrials.gov for drugs approved in 2022.

## Introduction

The 21st Century Cures Act, enacted in 2017, gave the US Food and Drug Administration (FDA) greater flexibility to apply evidence-based standards for novel drug approvals.^1^ To evaluate current practice, we summarize evidence supporting the 37 drugs approved in 2022.

## Methods

Using the FDA Novel Drug Approvals website, in a cross-sectional design, we examined 2022 approvals of all novel drugs that had not been previously approved for any indication. The study was conducted from August 1, 2022, to January 1, 2023. For each approved medication, all information in the ClinicalTrials.gov listing was downloaded, compiled into a comprehensive spreadsheet, and analyzed using SPSS, version 28.^2^ The study was certified as exempt by Oregon State University because it did not include human participants. We followed the STROBE reporting guideline. We coded the number of trials used for each approval, the total number of studies registered in ClinicalTrials.gov, the number of completed studies and those reporting results, and whether results were posted on ClinicalTrials.gov before the approval (eAppendix 1 and eAppendix 2 in Supplement 1).

## Results

The 37 drugs approved in 2022 were evaluated in 413 studies (mean [SD], 11.19 [13.04] studies per product; range, 1-68). A total of 227 studies (55%) were classified as randomized; 87 (21%) used single-group designs. Most studies (79%) received industry sponsorship, less than 1% were sponsored by the National Institutes of Health, and 2% represented a collaboration between the National Institutes of Health and a nonindustry source (Table).

**Table.** Summary of Frequencies and Percentages From 413 Studies Used to Evaluate the 37 Novel Drugs Approved by the Food and Drug Administration in 2022

Variable	Frequency (%)
Industry sponsored	326 (79)
Randomized clinical trials	227 (55)
Single group design	87 (21)
Completed studies	165 (40)
Results posted	103 (25)
Result posted after approval^a^	24 (23)

^a^ Based on posting of results from 103 studies.

Twenty-four drugs (65%) were approved based on a single study. Only 4 drugs (abrocitinib, oteseconazole, xenon Xe 129 hyperpolarized, and tirzepatide) were approved based on 3 or more studies (Figure). Among the 413 studies available for analysis, 165 (40%) were completed a mean of 8.4 (60.6) months before approval. Results were posted for 103 studies (25%) and, in 24 (6%) studies, results were first posted within 6 months after approval.

**Figure.**
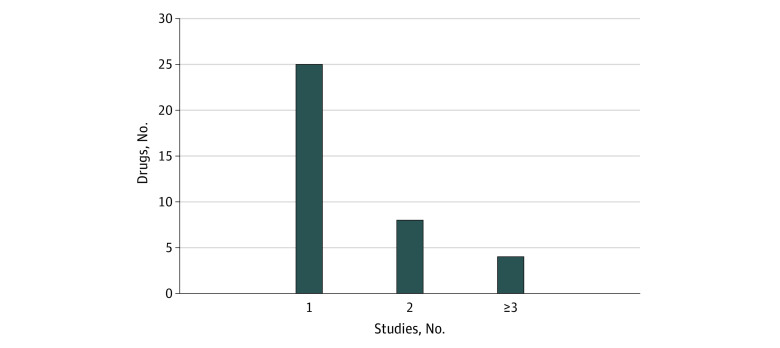
Frequency of Products Approved by the US Food and Drug Administration in 2022 on the Basis of 1, 2, or 3 or More Studies

## Discussion

Most of these 415 studies evaluating the 37 drugs approved in 2022 were sponsored by industry and 25% of the results have become publicly available. Only 55% of studies evaluating drugs approved in 2022 were randomized clinical trials, although most approvals were justified by randomized clinical trial data.

Our results highlight a trend toward less rigorous standards for novel drug approvals that has evolved over the past few decades.^1^ In contrast to 2016, when 4 of 20 products (20%) were approved based on a single trial, single studies justified 65% of the 2022 approvals. In 2016, 55% of products were approved based on 3 or more studies, in comparison with 11% in 2022.^1^

Our results are consistent with other reports. Zhang and associates^3^ also reported systematic decreases in the number of trials used for approvals. Piller^4^ observed that approximately 67% of studies from the 184 sponsor organizations with at least 5 trials failed to report their results in ClinicalTrials.gov, a number similar to our observations for 2022. Our finding that only 25% reported results in ClinicalTrials.gov is lower than the 50% reported by Zarin et al^5^ or Nelson et al,^6^ who found posting rates were about 39%. Federal efforts to increase reporting appear to have only small effects.

Limitations of this study include likely reporting and misclassification errors and truncation of the reporting period. The analysis was completed at the beginning of 2023, and it is possible that with additional time, other results would have been reported. Furthermore, our observation of a lower percentage of trials with results reported in ClinicalTrials.gov may reflect our focus on new approvals. Other studies had a longer trajectory, allowing more time for results to become public.

New drug approvals in 2022 appeared to be based on fewer studies than before passage of the 21st Century Cures Act. We believe consumers deserve access to the full range of evidence for the drugs they are considering, not just from the selected studies released to the public.

## Notes

Supplement 1. **eAppendix 1.** Steps for Downloading Data from FDA and from ClinicalTrials.gov **eAppendix 2.** Selected SPSS Code for Analyses

Supplement 2.
Data Sharing Statement
