# Supplementary material for: Review of Evidence Supporting 2022 US Food and Drug Administration Drug Approvals
Source: JAMA Netw Open. 2023 Aug 8;6(8):e2327650. doi: 10.1001/jamanetworkopen.2023.27650 (PMC10410475; doi:10.1001/jamanetworkopen.2023.27650)
Supplement: Supplement 1. — eAppendix 1. Steps for Downloading Data from FDA and from ClinicalTrials.gov eAppendix 2. Selected SPSS Code for Analyses [file jamanetwopen-e2327650-s001.pdf]

Supplementary Online Content

Kaplan RM, Koong AJ, Irvin V. Review of evidence supporting 2022 US Food and Drug Administration drug approvals. *JAMA Netw Open*. 2023;6(8) e2327650. doi:10.1001/jamanetworkopen.2023.27650

eAppendix 1. Steps for Downloading Data from FDA and from ClinicalTrials.gov

eAppendix 2. Selected SPSS Code for Analyses

This supplementary material has been provided by the authors to give readers additional information about their work.

© 2023 Kaplan RM et al. JAMA Network Open.

eAppendix 1. Steps for Downloading Data from FDA and from ClinicalTrials.gov

1. Go to FDA Webpage for "Novel Drug Approvals for 2022"
2. Scroll down to summary table at the bottom of the page.
3. For each drug, record the drug name, the active ingredient, the approval date, and the approved FDA use as of the date of drug approval.
4. Use the "drug trials snapshot" to determine the number of trials used in the approval. If the snapshot is not available, search for the FDA press release to obtain the same information.
5. Go to clinicaltrials.gov and search for the drug using the active ingredient in the "other terms" field and the clinical indication in the "condition or disease" field.
6. Inspect the results to make sure they appear reasonable.
7. Use the "download" button at the upper right portion of the screen.
8. From the download, use the number of studies pulled down, and select "all studies". From the "select table columns", use "all available columns". For Select file format, chose "Tab separated values"
9. The downloaded file should be in text format. It can be copied directly into excel.

© 2023 Kaplan RM et al. JAMA Network Open.

eAppendix 2.
Selected SPSS Code for Analyses

```
GET
  FILE='/Users/Kaplan/Library/CloudStorage/Dropbox/FDA Studies
March 2023/FDA 2022 data 3-27-2023.sav'.
DATASET NAME DataSet5 WINDOW=FRONT.
DATASET ACTIVATE DataSet1.
FREQUENCIES VARIABLES=CompleteBefore DrugName
Numberoftrialsinapproval Status SponsorCollaborators
    FundedBys StudyType StudyDesigns FirstPosted
ResultsFirstPosted V30 V31 V32
  /STATISTICS=MINIMUM MAXIMUM MEAN MEDIAN SKEWNESS SESKEW
KURTOSIS SEKURT
  /ORDER=ANALYSIS.
GET DATA
  /TYPE=XLSX
  /FILE='/Users/Kaplan/Library/CloudStorage/Dropbox/JAMA Open
Appendix 1.xlsx'
  /SHEET=name 'Sheet1'
  /CELLRANGE=FULL
  /READNAMES=ON
  /DATATYPEMIN PERCENTAGE=95.0
  /HIDDEN IGNORE=YES.
EXECUTE.
DATASET NAME DataSet4 WINDOW=FRONT.
  /ORDER=ANALYSIS.
FREQUENCIES VARIABLES=CompletedApproval StudyDesigns
  /ORDER=ANALYSIS.
FREQUENCIES VARIABLES=SponsorCollaborators
  /ORDER=ANALYSIS.
GGRAPH
  /GRAPHDATASET NAME="graphdataset"
    VARIABLES=CompletedApproval[LEVEL=scale]
    MISSING=LISTWISE REPORTMISSING=NO
  /GRAPHSPEC SOURCE=VIZTEMPLATE(NAME="Histogram with Normal
Distribution"[LOCATION=LOCAL]
    MAPPING( "x"="CompletedApproval"[DATASET="graphdataset"]))
    VIZSTYLESHEET="Traditional"[LOCATION=LOCAL]
    LABEL='HISTOGRAM WITH NORMAL DISTRIBUTION:
CompletedApproval'
    DEFAULTTEMPLATE=NO.
```

© 2023 Kaplan RM et al. JAMA Network Open.
