# Supplementary material for: Review of Evidence Supporting 2022 US Food and Drug Administration Drug Approvals
Source: JAMA Netw Open. 2023 Aug 8;6(8):e2327650. doi: 10.1001/jamanetworkopen.2023.27650 (PMC10410475; doi:10.1001/jamanetworkopen.2023.27650)
Supplement: Supplement 2. — Data Sharing Statement [file jamanetwopen-e2327650-s002.pdf]

Data Sharing Statement

Kaplan. Review of Evidence Supporting 2022 US Food and Drug Administration Drug Approvals. *JAMA Netw Open*. Published August 08, 2023.
doi:10.1001/jamanetworkopen.2023.27650

Data
Data available: Yes
Data types: Data (not involving human participants), Data dictionary
How to access data: We will provide access to the data on a Stanford University website
When available: With publication

Supporting Documents
Document types: Statistical/analytic code
How to access documents: The data will and supporting code will be posted on a Stanford University website
When available: With publication

Additional Information
Who can access the data: The data will be made available to anyone.
Types of analyses: Data can be used for any purposeWith
Mechanisms of data availability: With a signed data agreement
Any additional restrictions: None
